# Supplementary material for: Scalable computation of anisotropic vibrations for large macromolecular assemblies
Source: Nat Commun. 2024 Apr 24;15:3479. doi: 10.1038/s41467-024-47685-8 (PMC11043083; doi:10.1038/s41467-024-47685-8)
Supplement: Supplementary file 1 — Supplementary Information [file 41467_2024_47685_MOESM1_ESM.pdf]

Supplementary Information

In support of the article "Scalable computation of anisotropic vibrations for large macromolecular assemblies" by Jordy Homing Lam, Aiichiro Nakano and Vsevolod Katritch.

Table of Content *Page*

- Supplementary Methods 2
- Supplementary Figures 10

Supplementary Methods

March 20, 2024

In support of the article "Scalable computation of anisotropic vibrations for large macromolecular assemblies" by Jordy Homing Lam, Aiichiro Nakano and Vsevolod Katritch. It contains the pseudocodes for the following algorithms:

Algorithm 1 3-D Reverse Cuthill McKee (3DRCM)

Algorithm 2 Modified Gram-Schmidt Vector (MGSV)

Algorithm 3 Iterative Classical Gram-Schmidt (ICGS)

Algorithm 4 p-step Lanczos Factorization (PLF)

Algorithm 5 Implicitly Restarted Lanczos Method (IRLM)

Algorithm 6 Thick Restart Lanczos Method (TRLM)

Algorithm 7 Jacobi-Davidson Method (JDM)

Algorithm 8 Chebyshev-Davidson Method (CDM)

Algorithm 9 Chebyshev Filtered Matrix Vector Product (ChebAv)

1 3-D Reverse Cuthill McKee

A Reverse Cuthill McKee algorithm supplemented with a k-D tree data structure where k=3. Analysis of a k-D tree can be found in the work of 'Bentley, J. L. Multidimensional binary search trees used for associative searching. Commun. ACM 18, 509–517 (1975)'. An empty 1-d array of length l is notated as 0^l. Time complexity marked in comment.

Algorithm 1 3-D Reverse Cuthill McKee

```
procedure 3DRCM(X ∈ ℝ^{N×3}, R_C ∈ ℝ^1)
                                                  ▷ (a) Prepare a 3-D tree O(NlogN)
    3dTree ← KdTree(X)
                                                  ▷ (b) Find peripheral node O(N × 3N^{2/3})
    Initialize D = 0^N                            ▷ Degrees.
    for i = 0, ..., N − 1 do
        |𝒩(X_i)| = 3dTree(R_C, X_i)
        D[i] ← |𝒩(X_i)|
    Inds = Argsort(D) ; IndsRev = Argsort(Inds)   ▷ Quicksort.
    m = max(D)
                                                  ▷ (c) Breadth-first loop O(3N^{5/3} + 2cm^2N)
    Initialize L = 0^N ; D̃ = 0^m; n = 0
    for z = 0, ..., N − 1 do
        if Inds[z] == −1 then
            continue
        L[n] ← Inds[z]; n += 1
        Inds[IndsRev[Inds[z]]] ← −1               ▷ Indicate visited.
        Level_s = n − 1; Level_t = n
        while Level_s < Level_t do
            for ĩ = Level_s, ..., Level_t − 1 do
                i ← L[ĩ] ; n_old = n
                𝒩(X_i) = 3dTree(R_C, X_i)         ▷ Neighbors in R_C.
                for j = 0, ..., |𝒩(X_i)| − 1 do
                    j̃ = 𝒩(X_i)[j]
                    if Inds[IndsRev[j̃]] == −1 then
                        continue
                    Inds[IndsRev[j̃]] ← −1
                    L[n] ← j ; n += 1
                l_s = 0
                for k = n_old, ..., n − 1 do      ▷ Insertion Sort.
                    D̃[l_s] ← D[L[k]] ; l_s += 1
                for k = 1, ..., l_s − 1 do
                    d̃ ← D̃[k]
                    l̃ ← L[n_old + k]
                    l_t ← k
                    while l_t > 0 and d̃ < D̃[l_t − 1] do
                        D̃[l_t] ← D̃[l_t − 1]
                        L[n_old + l_t] ← L[n_old + l_t − 1]; l_t −= 1
                    D̃[l_t] ← d̃
                    L[n_old + l_t] ← l̃
            Level_s ← Level_t ; Level_t ← n
                                                  ▷ (d) Reverse
    return L[:: −1]
```

2 Modified Gram-Schmidt Vector

A Modified Gram-Schmidt (MGS) algorithm to orthogonalize a vector u against the basis $V = [v_0, ..., v_m]$.

Algorithm 2 Modified Gram-Schmidt Vector

procedure MGSV($u \in \mathbb{R}^n; V \in \mathbb{R}^{m \times n}$)
    for $i = 0, ..., m-1$ **do**
        $u \leftarrow u - (u^\top v_i) v_i$
    return u

3 Iterative Classical Gram-Schmidt

An Iterative Classical Gram-Schmidt (ICGS) algorithm to orthogonalize a vector u against the basis $V = [v_0, ..., v_m]$. A break is triggered when the deflation is revoked.

Algorithm 3 Iterative Classical Gram-Schmidt

procedure ICGS($u \in \mathbb{R}^n; V \in \mathbb{R}^{m \times n}$)
    $r_0 = ||u||_2$
    for $i_{iter} = 1, 2, 3$ **do**
        $u \leftarrow u - VV^\top u$
        $r_1 = ||u||_2$
        if $r_1 > r_0/2$ **then**
            Break
        $r_0 \leftarrow r_1$
    if $r_1 \leq r_0/2$ **then**
        Warning! Loss of orthogonality
    return u

4 p-step Lanczos Factorization

A p-step Lanczos Factorization (PLF) to produce p basis between index j_s and j_t. Algorithm 4 PLF is where a polynomial filter can be applied, for example, in Algorithm 8 CF with a first-kind Chebyshev polynomial. Step 9 and 10 are optional; a proof of the spectrum bound can be found in 'Zhou et al, Bounding the spectrum of large Hermitian matrices, Linear Algebra and its Applications 435(3), 2011, p.480-493, https://doi.org/10.1016/j.laa.2010.06.034'. In our case, the lower bound is well known.

Algorithm 4 p-step Lanczos Factorization

1: **procedure** PLF($A \in \mathbb{R}^{n \times n}, V \in \mathbb{R}^{(m+1) \times n}, \alpha \in \mathbb{R}^m, \beta \in \mathbb{R}^m, j_s = 0, j_t = m$)
2:     **for** $j = j_s, ..., j_t - 1$ **do**
3:         $r = Av_j$ ▷ $r = p(A, v_j, a, b)$ if filter in use.
4:         $\alpha_j \leftarrow v_j^\top r$
5:         $r \leftarrow r - \alpha_j v_j$
6:         $r \leftarrow MGSV(r, V[:j]); r \leftarrow MGSV(r, V[:j])$ ▷ Full Reorthogonalization (FRO).
7:         $\beta_j \leftarrow ||r||_2$
8:         $v_{j+1} = r/\beta_j$
9:     $T[j,j] = \alpha; T[j, j-1] = T[j-1, j] = \beta$
10:     Solve $TQ = QD$ ▷ Spectrum upper bound as $D_{max} + \beta_j ||e_j^\top Q_j||_\infty$
11:     **return** V, α, β

5 Implicitly Restarted Lanczos Method

An Implicitly Restarted Lanczos Method (IRLM) to compute k smallest eigenpairs. This algorithm calls Algorithm 2 MGSV and Algorithm 4 PLF. At max, 15000 restarts were allowed.

Algorithm 5 Implicitly Restarted Lanczos Method

procedure IRLM($A \in \mathbb{R}^{n \times n}, V \in \mathbb{R}^{(m+1) \times n}, \alpha \in \mathbb{R}^m, \beta \in \mathbb{R}^m, \epsilon = 1e - 8$)
  Initialize $v_0 \leftarrow v_{rand}/||v_{rand}||_2$
  $j_s = 0$
  for $i_{iter} = 1, ..., 15000$ **do**
    ▷ (a) Initial p-steps Lanczos Factorization
    PLF(A,V,α,β, $j_s = j_s$, $j_t = m$)
    ▷ (b) Implicit Shift
    $S, W = eig(\alpha, \beta[: m - 1])$
    $\theta = Sort(Diag(S))[:: -1][: (m - k)]$
    $\tilde{\beta} = \beta_{k+p-1}$
    $Q = I$
    for $i = 0...p - 1$ **do**
      $T = Tridiag(\alpha, \beta[: m - 1])$
      $\tilde{Q}\tilde{R} = T - \theta_i I$
      $T \leftarrow \tilde{Q}^\top T \tilde{Q}$
      $\alpha, \beta \leftarrow T$
      $Q \leftarrow Q\tilde{Q}$
    $\beta \leftarrow [\beta, \tilde{\beta}]$
    ▷ (c) Implicit Restart
    $\sigma = Q[k - 1, m - 1]$
    $V[: k] \leftarrow Q[: k, :]V[: m, :]$
    $v_k \leftarrow \beta_{k-1} Q[k, :]V[: m, :] + \sigma\beta_{m-1} v_m$
    $\beta_{k-1} \leftarrow ||v_k||_2$
    $v_k \leftarrow v_k/\beta_{k-1}$
    $v_k \leftarrow MGSV(v_k, V[: k]); v_k \leftarrow MGSV(v_k, V[: k])$
    $v_k \leftarrow v_k/||v_k||_2$
    $j_s \leftarrow k$ ▷ Reset the PLF
    ▷ (d) Estimate Convergence
    if $|\beta_{k-1}| < \epsilon$ **then**
      Break
  $S, W = eig(Tridiag(\alpha[: k], \beta[: k - 1]))$
  return $S[: k], W^\top V[: k, :]$

6 Thick Restart Lanczos Method

A Thick Restart Lanczos Method to compute the k smallest eigenpairs. This algorithm calls Algorithm 2 MGSV and Algorithm 4 PLF. Algorithm 4 PLF is where a filter can be applied through the matrix-vector multiplication, for example, in Algorithm 9, a filter is constructed using first-kind Chebyshev polynomial basis. At max, 15000 restarts were allowed.

Algorithm 6 Thick Restart Lanczos Method

procedure TRLM($A \in \mathbb{R}^{n\times n}, V \in \mathbb{R}^{(m+1)\times n}, \alpha \in \mathbb{R}^m, \beta \in \mathbb{R}^m, \epsilon = 1e-12$)
    Initialize $v_0 \leftarrow v_{rand}/||v_{rand}||_2$
    $j_s = 0; b = 0; S = 0; r = v_0$
    for $i_{iter} = 1, ..., 15000$ **do**
        ▷ (a) Reinitialize
        $\beta[: k] \leftarrow 0$
        $\alpha[: k] \leftarrow Diag(S)$
        $r \leftarrow MGSV(r, V[: k])$
        $r \leftarrow r/||r||_2$
        $v_k \leftarrow r$
        ▷ (b) p-step Lanczos Factorization, filter inside.
        PLF(A,V,α,β, $j_s = j_s$, $j_t = m$)
        ▷ (c) Thick Restart
        $T = Tridiag(\alpha, \beta[: m-1]); T[k, : k] \leftarrow b; T[: k, k] \leftarrow b$
        $S, W = eig(T)$
        $W \leftarrow W[: m, Argort(Diag(S))[: k]]$
        $S \leftarrow Diag(S)[Argort(Diag(S))[: k]]$
        $V[: k, :] \leftarrow W^\top V$
        $b = \beta_{m-1} W[m-1, : k]$
        $j_s \leftarrow k$ ▷ Reset the PLF
        ▷ (d) Check Convergence
        if $||b||_2 < \epsilon$ **then**
            Break
    return S, V

7 Jacobi-Davidson Method

A Jacobi-Davidson Method to compute k smallest eigenpairs. This algorithm calls Algorithm 2 MGSV and Algorithm 3 ICGS. The correction z is approximated with Generalized Minimal Residual Iteration (GMRES) routine provided in CuPy; unless otherwise stated, the iteration is stopped if residual error in GMRES reach 1e-6 or the number of iterations exceeds 20. Note that this can be replaced by a Minimal Residual Iteration (MIRES) routine, which processes symmetric matrices. j_s counts towards the desired number of converged Ritz pair k. Some preconditioners (e.g. ILU(k)) were considered in solving the correction equation, but their size and density become prohibitive as n increases; trivial preconditioner e.g. $diag(A)^{-1}$ does not improve performance.

Algorithm 7 Jacobi-Davidson Method

procedure JDM($A \in \mathbb{R}^{n\times n}, \epsilon = 1e-12, k = 64$)
  Initialize $v_0 = v_{rand}/||v_{rand}||_2$
  $G = v_0^\top A v_0$
  $V = [v_0,]$
  $Q = [0^n,]; \Lambda = []$
  $j_s = 0$

  for $i_{iter} = 1, ..., 15000$ **do**
    $S, W = eig(G)$
    while True **do**
      ▷ (a) Get Residual
      $u = VW[:, 0]$; $\theta = S[0, 0]$
      $r = Au - \theta u$
      $\sigma = \theta$ ▷ Propose shift.
      $\tilde{Q} = [Q, u]$

      if $(||r||_2 > \epsilon) \cup ((dim(S)[0] <= 1) \cap (j_s \mathrel{!}= k-1))$ **then** ▷ Non-convergence.
        Break.
      ▷ (b) Update Projections
      $\Lambda \leftarrow [\Lambda, \theta]$
      $Q \leftarrow \tilde{Q}$
      $V \leftarrow VW[:, 1:j]; S \leftarrow S[1:j, 1:j]$
      $G \leftarrow S; W \leftarrow I$
      $j_s \mathrel{+}= 1$
      if $j_s == k$ **then**
        Return Λ, Q ▷ All converged.

    if $dim(S)[0] == 2k$ **then** ▷ (c) Restart if workspace is full
      $V \leftarrow VW[:, 0:k]; S \leftarrow S[0:k, 0:k]$
      $G \leftarrow S; W \leftarrow I$
    ▷ (d) Correction equation with GMRES.
    $(I - uu^\top)(A - \theta I)(I - uu^\top)z = -r$
    $z \leftarrow MGSV(\tilde{Q}, z)$
    $z \leftarrow ICGS(V, z)$; $z \leftarrow ICGS(V, z)$
    $z \leftarrow z/||z||_2$
    ▷ (e) Update Projections
    $\tilde{z} = Az$
    $V \leftarrow [V, z]$
    $G \leftarrow [G, V^\top \tilde{z}; \tilde{z}^\top V, z^\top \tilde{z}]$
  ▷ (f) Guard not all converged.
  return Λ, Q

8 Chebyshev-Davidson Method

A Chebyshev-Davidson Method to compute k smallest eigenpairs. This algorithm calls Algorithm 2 MGSV and Algorithm 3 ICGS. λ_{min} is the lower bound of spectrum. λ_{max} is upper bound of the spectrum obtained from Algorithm 4 PLF. θ_s is the 'squeezing' moving lower bound.

Algorithm 8 Chebyshev-Davidson Method

procedure CDM($A \in \mathbb{R}^{n\times n}, \lambda_{min}, \lambda_{max}, \epsilon = 1e-12, k = 64,$)
    Initialize $v_0 = v_{rand}/||v_{rand}||_2$
    $G = v_0^\top A v_0$
    $V = [v_0,]$
    $Q = [Av_0,]; \Lambda = []$
    $j_s = 0; j = 1$
    $u = V[:, j_s]$
    $\theta_s = (\lambda_{max} + G[0, 0])/2$

    for $i_{iter} = 1, ..., 15000$ **do**
        $z = p(A, u, \theta_s, \lambda_{max})$ ▷ (a) Low-pass Chebyshev filter
        $z \leftarrow MGSV(\tilde{Q}, z)$
        $z \leftarrow ICGS(V, z)$
        $z \leftarrow z/||z||_2$
        ▷ (b) Update Projections
        $\tilde{z} = Az$
        $V[:, j] \leftarrow z$
        $Q[:, j] \leftarrow \tilde{z}$
        $G[j, j_s : j] \leftarrow Q[:, j]^\top V[:, j_s : j+1]$
        $G[j_s : j, j] \leftarrow G[j, j_s : j]^\top$
        $S, W = eig(G)$
        ▷ (c) Restart if workspace is full
        $j_r = j + 1$
        if $j + 1 >= 2k$ **then**
            $j_r = max(j_s + 1, k + 5, min(k + j_s, 2k - 5))$
        $V[:, j_s : j_r] \leftarrow V[:, j_s : j+1]W[:, 0 : j_r - j_s + 1]$
        $Q[:, j_s : j_r] \leftarrow Q[:, j_s : j+1]W[:, 0 : j_r - j_s + 1]$
        $G[j_s : j_r, j_s : j_r] \leftarrow S[0 : j_r - j_s + 1, 0 : j_r - j_s + 1]; W \leftarrow I$
        ▷ (d) Get Residual
        $\theta = S[0]$
        $r = Q[:, j_s] - \theta V[:, j_s]$
        $u = V[:, j_s]$ ▷ Next u
        if $||r||_2 < \epsilon$ **then**
            $\Lambda \leftarrow [\Lambda, \theta]; j_s += 1$
            if $j_s >= k$ **then**
                Return Λ, Q ▷ All converged.
            $u = V[:, j_s - 1]$ ▷ Next u
        $j = j_r$
        ▷ (e) Update moving lower bound
        $\theta_s = max(S_{median}, \lambda_{min})$
        ▷ (f) Guard not all converged.
    return Λ, Q

9 Chebyshev Filtered Matrix Vector Product

Matrix vector product filtered on the basis of first-kind Chebyshev polynomial. $p(t) = \sum_{j=0}^{j=M} \kappa_j T_j(t)$. Note that (1) κ_j is a set of user-defined coefficients e.g. the scaled coefficients in equation 28 of the main text. (2) In Algorithm 8 CDM, only the M-th degree polynomial is considered.

Algorithm 9 Chebyshev Filtered Matrix Vector Product

procedure CHEBAV($A \in \mathbb{R}^{n\times n}, v \in \mathbb{R}^{n\times 1}, a, b$)
  $e =: (b-a)/2; c =: (b+a)/2$
  $y = \kappa_0 v$
  $v_+ = \kappa_1(Av - cv)/e$
  $y \mathrel{+}= \kappa_1 v_+$
  $v_- = v; v \leftarrow v_+;$
  for $j = 2, ..., M$ **do**
    $v_+ = 2/e(Av - cv) - v_-$
    $y \mathrel{+}= \kappa_j v_+$
    $v_- \leftarrow v; v \leftarrow v_+$
  return y

Supplementary Figures

In support of the article “Scalable computation of anisotropic vibrations for large macromolecular assemblies” by Jordy Homing Lam, Aiichiro Nakano and Vsevolod Katritch.

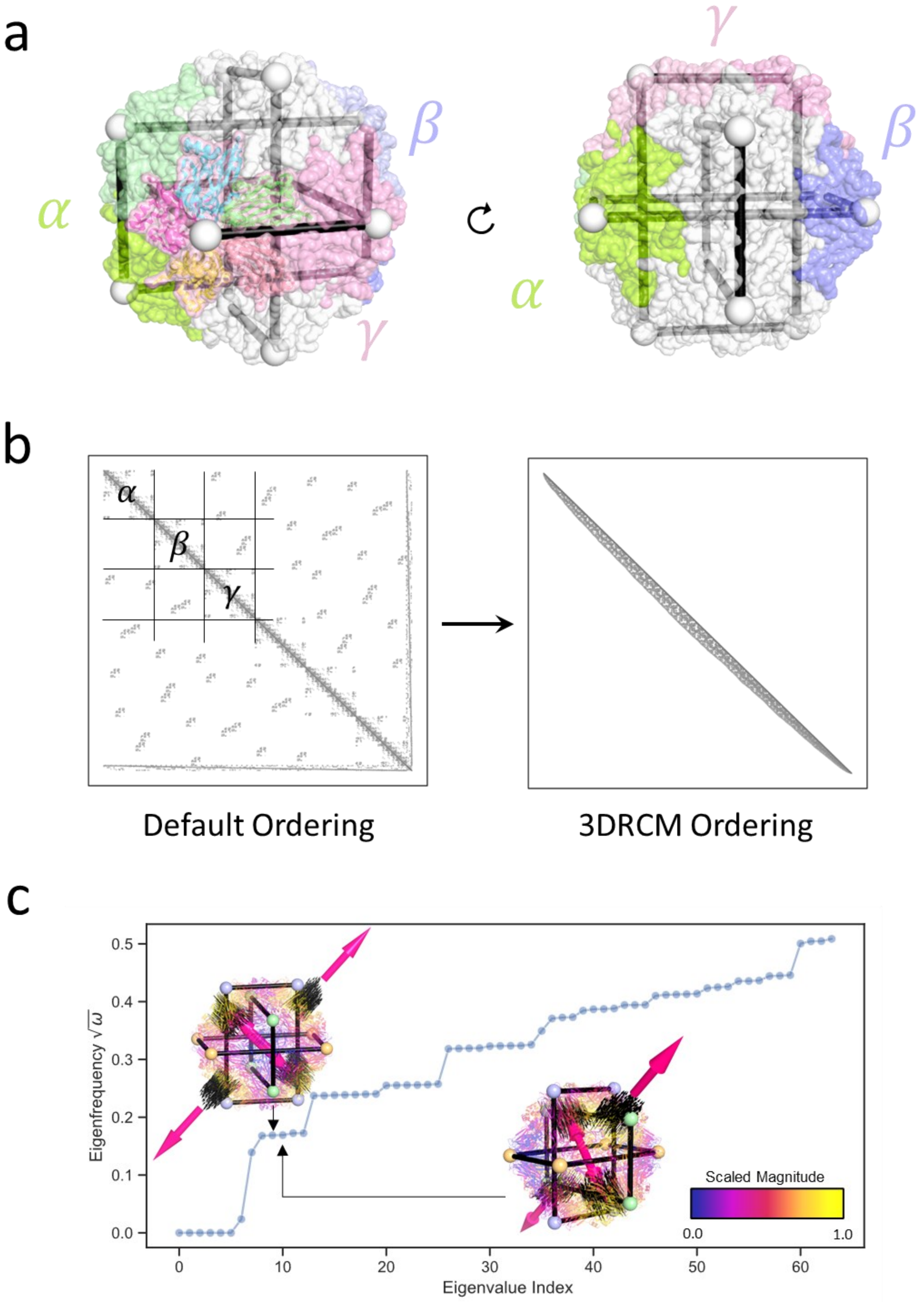

a
α
β
γ
b
α
β
γ
Default Ordering
3DRCM Ordering
c
Eigenfrequency $\sqrt{\omega}$
Eigenvalue Index
Scaled Magnitude
0.0
1.0

Supplementary Figure 1. Layout of the Hessian Matrix with and without permutation of atom ordering for a case study on the chimeric Sesbania mosaic virus coat protein (PDBID: 4Y5Z). The protein structure is a homo-60-mer made of 12 sets of pentamers arranged as an icosahedron with apparent symmetry. (a) Overall spatial distribution of the pentamers in default sequential order. Segment α, β , and γ were colored in green, blue and pink respectively. Segment α consist of chain A,B,C,D,E,F,G,H,I and J. Segment β consist of chain K,L,M,N,O,P,Q,R,S and T. Segment γ consist of chain U, V, W, X, Y, Z, a, c, e and g. The three orthogonal golden rectangles were shown in black with vertices in white sphere. The rest of the macromolecule is shown as a grey surface. A pentamer is displayed as cartoons in segment γ. (b) The Hessian matrix in default ordering (left) and the lower triangle of the Hessian matrix in 3-Dimensional Reverse Cuthill McKee (3DRCM) ordering (right). The Hessian matrix in default ordering has a much longer bandwidth everywhere than the Hessian matrix in 3DRCM ordering; the last few rows and columns in the default ordering refers to water and ions. In the default ordering, it is also clear that segment α do not interact with segment β, but segment γ interacts with both α and β. (c) The first 64 eigenfrequency of the macromolecule, the eigenfrequency is the square root of eigenvalue ω. Note that the macromolecule is asymmetric due to the inconsistent presence of waters. Inset shows the mode shapes from two apparently degenerate modes. The black arrows are the displacement field of 1000 atoms randomly chosen from those in the top 90% quantile of vibration magnitude in the eigenvector. The arrow in magenta indicates an average direction for local clusters of the displacement field. The vertices belonging to the same golden rectangle is displayed in the same color.

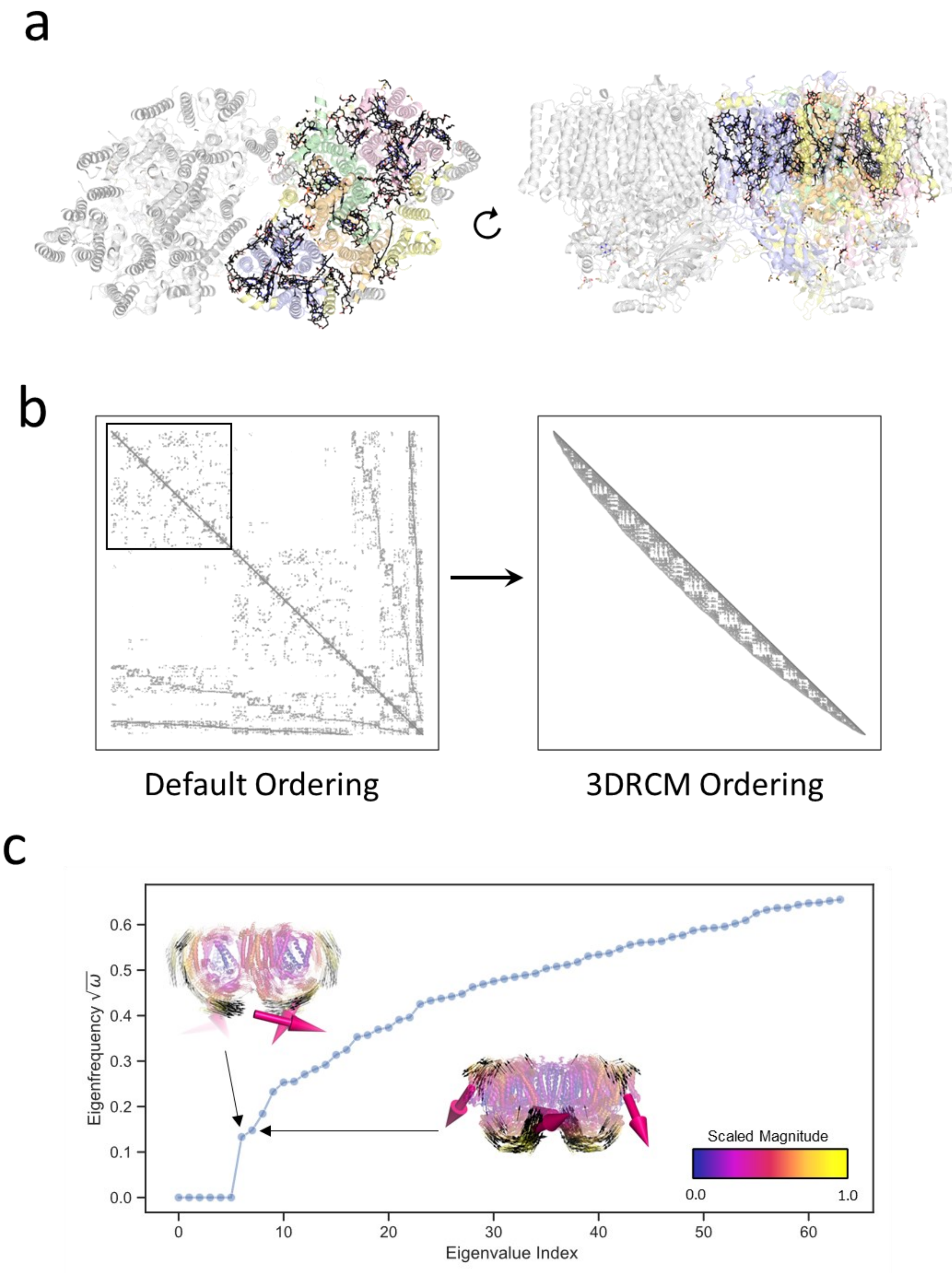
a
b
Default Ordering
3DRCM Ordering
c
Eigenfrequency $\sqrt{\omega}$
Eigenvalue Index
Scaled Magnitude
0.0
1.0
0.0
0.1
0.2
0.3
0.4
0.5
0.6
0
10
20
30
40
50
60

Supplementary Figure 2. Layout of the Hessian Matrix with and without permutation of atom ordering for a case study on the PsbM-deletion mutant of photosystem II (PDBID: 5H2F). The macromolecular complex is made of cofactors, lipids, water and protein chains. (a) Overall spatial distribution of the chemicals intercalated in multiple protein chains in default sequential order (green, blue, pink, orange, yellow). Chemicals were shown in black. Only the right half of the structure is colored, the rest of the macromolecule is shown as a grey surface. (b) The Hessian matrix in default ordering (left) and the lower triangle of the Hessian matrix in 3-Dimensional Reverse Cuthill McKee (3DRCM) ordering (right). The Hessian matrix in default ordering has a much longer bandwidth everywhere than the 3DRCM ordering. The top left square highlighted in the Hessian matrix of the default ordering correspond to that of the right half proteins. Note that the macromolecule is asymmetric due to slight difference in chemicals intercalated. (c) The first 64 eigenfrequencies of the macromolecule, the eigenfrequency is the square root of eigenvalue ω. Inset shows the stacked mode shapes from each of the first two modes. The black arrows are the displacement field of 1000 atoms randomly chosen from those in the top 90% quantile of vibration magnitude in the eigenvector. The arrow in magenta indicates an average direction for local clusters of the displacement field.

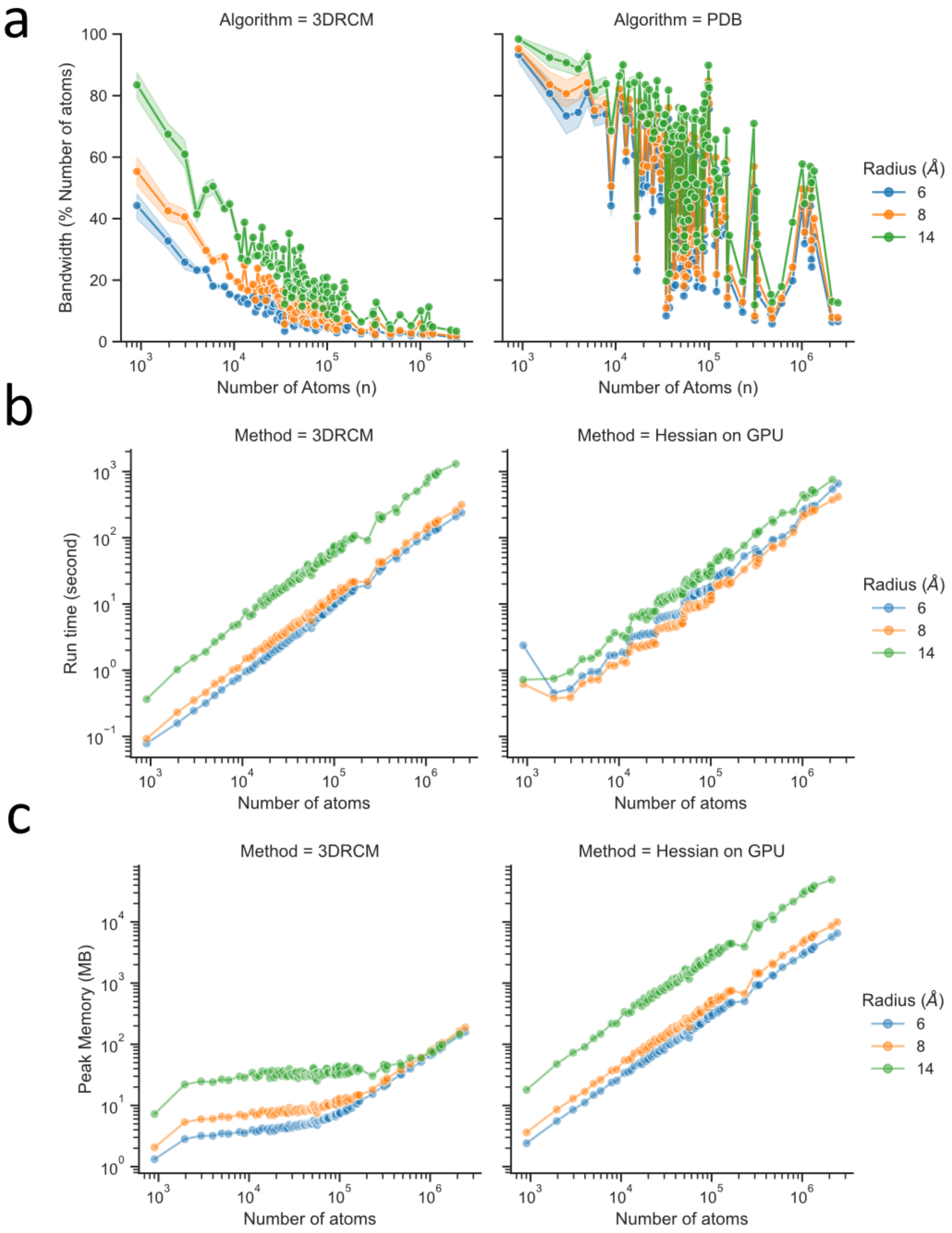
a
Algorithm = 3DRCM
Algorithm = PDB
Bandwidth (% Number of atoms)
Number of Atoms (n)
Radius (Å)
6
8
14
b
Method = 3DRCM
Method = Hessian on GPU
Run time (second)
Number of atoms
c
Method = 3DRCM
Method = Hessian on GPU
Peak Memory (MB)
Number of atoms

Supplementary Figure 3. Benchmarks on permutation and construction of the Hessian. Color code refers to radius R_C = 6 Å (blue),8 Å (orange) or 14 Å (green). (a) Mean bandwidth of a 100-atoms batch of Hessian in default Protein Data Bank (PDB) ordering and 3-Dimensional Reverse Cuthill McKee (3DRCM) ordering. To facilitate communication, the mean bandwidth reported here is the mean bandwidth in terms of atom slice (i.e., $N\times N$) of the ($N\times N\times 3\times 3$) Hessian tensor. The mean bandwidth is presented as the percentage of the total number of atoms N. The Hessian realized with 3DRCM permuted atom ordering is consistently less than 10% of N once there are more than 100 thousand atoms, whereas in the default PDB ordering it can fluctuate between 10% and 90% of N. The shade is the 95% confidence interval. (b) Run time of the algorithms. The reported time is wall-clock time. (c) Peak Memory of the algorithms. Note that for 3DRCM, the memory reported is in random access memory, whereas for the Hessian on GPU, the memory reported is the GPU memory. For R_C = 14 Å, 64-bit indexing were used throughout instead of 32-bit indexing to accommodate the larger amount of non-zero entries.

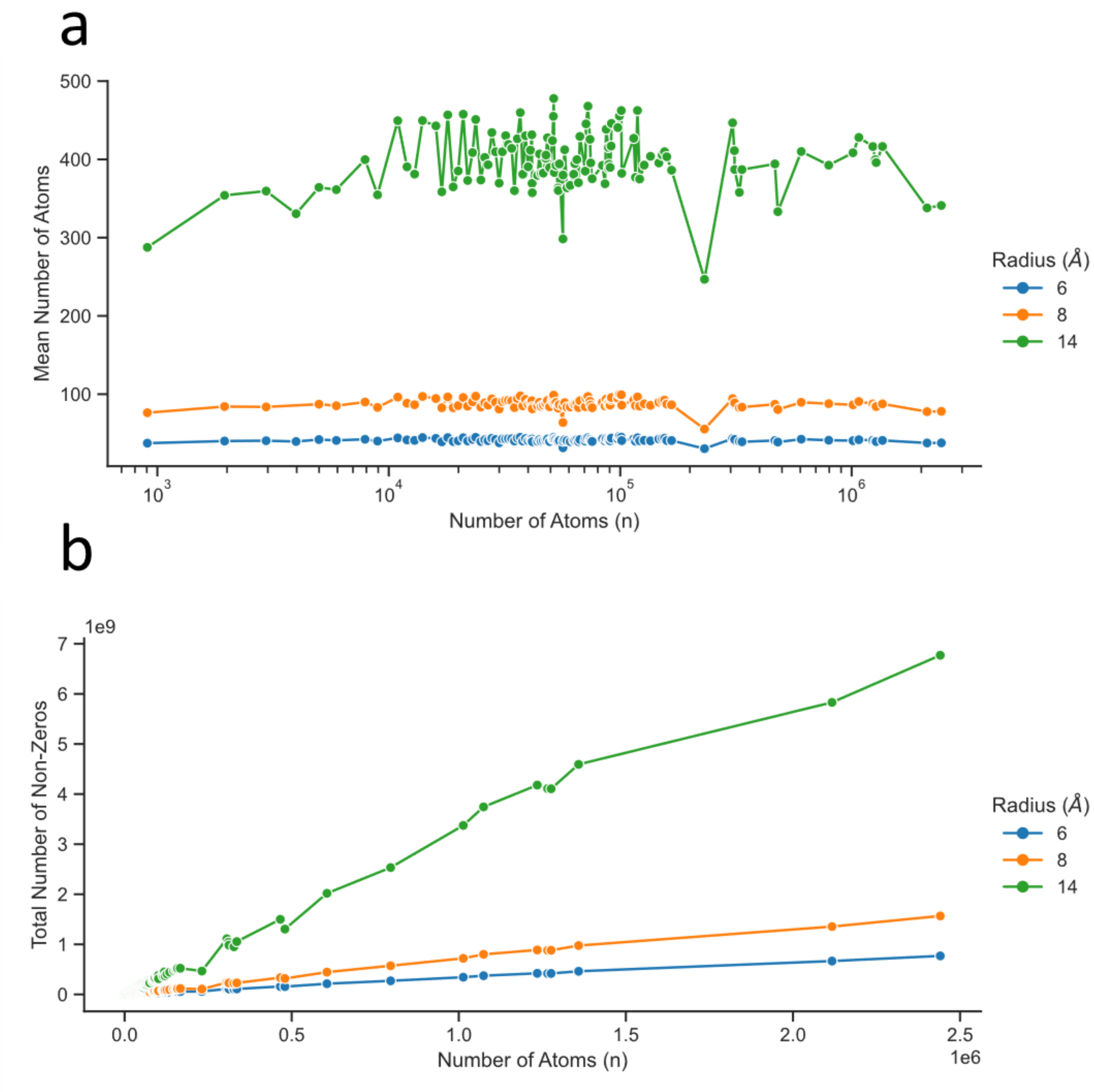

Supplementary Figure 4. Information on the benchmark dataset. The benchmark dataset has 116 macromolecules with size ranging from around 1000 atoms to around 2.4 million atoms. Color code refers to radius R_C = 6 Å (blue),8 Å (orange) or 14 Å (green). (a) Mean number of atoms within a sphere of radius R_C. (b) Total number of non-zero entries in the half Hessian matrix.

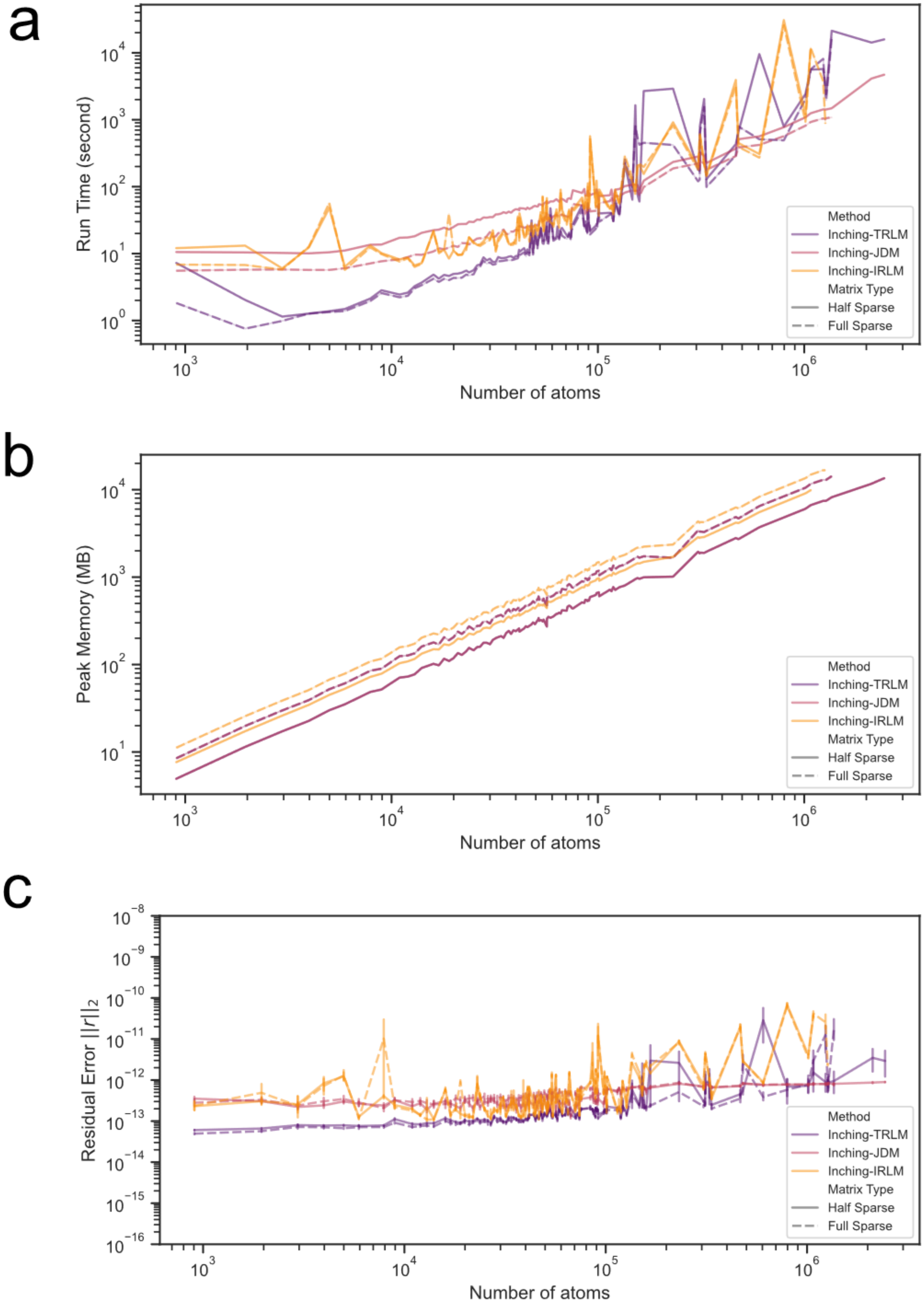
a
Run Time (second)
Number of atoms
Method
Inching-TRLM
Inching-JDM
Inching-IRLM
Matrix Type
Half Sparse
Full Sparse
b
Peak Memory (MB)
Number of atoms
Method
Inching-TRLM
Inching-JDM
Inching-IRLM
Matrix Type
Half Sparse
Full Sparse
c
Residual Error $||r||_2$
Number of atoms
Method
Inching-TRLM
Inching-JDM
Inching-IRLM
Matrix Type
Half Sparse
Full Sparse

Supplementary Figure 5. Comparison of the effect of accessing and storing the full Hessian matrix rather than the lower triangle on throughput, memory consumption and correctness in the benchmark dataset. For all methods, the first 64 eigenpairs were calculated with radius R_C = 8 Å. Methods tested includes 3 INCHING methods (i.e. INCHING-TRLM, INCHING-JDM, INCHING-IRLM) with only the lower triangle of the Hessian stored and accessed in dash line and with the full Hessian matrix stored and accessed in solid line. The methods in dash line with only the lower triangle of the Hessian stored were also displayed in Figure 2 of main text. In all methods, the storage format of the Hessian matrix is always "Sparse" meaning a Compressed Sparse Row (CSR) format is stored in double precision. INCHING-TRLM, INCHING-JDM, INCHING-IRLM are our implementation of the Thick Restart Lanczos Method (TRLM), Jacobi-Davidson Method (JDM) and Implicitly Restarted Lanczos Method (IRLM). (a) Benchmark on overall run time including Hessian realization and subsequent diagonalization. The run time is wall-clock time to complete all the calculation. (b) Benchmark on peak memory consumption. Also note that INCHING-TRLM-HalfSparse and INCHING-JDM-HalfSparse share very similar peak memory requirement. (c) Benchmark on residual error. The error bar presented is the 95% confidence interval (n=64) calculated from the residual error of all the eigenvalues of a macromolecular structure in the benchmark dataset. All programs were stopped if run time exceeds 48 hours. All INCHING programs were stopped if number of restarts exceeded 15000 rounds.

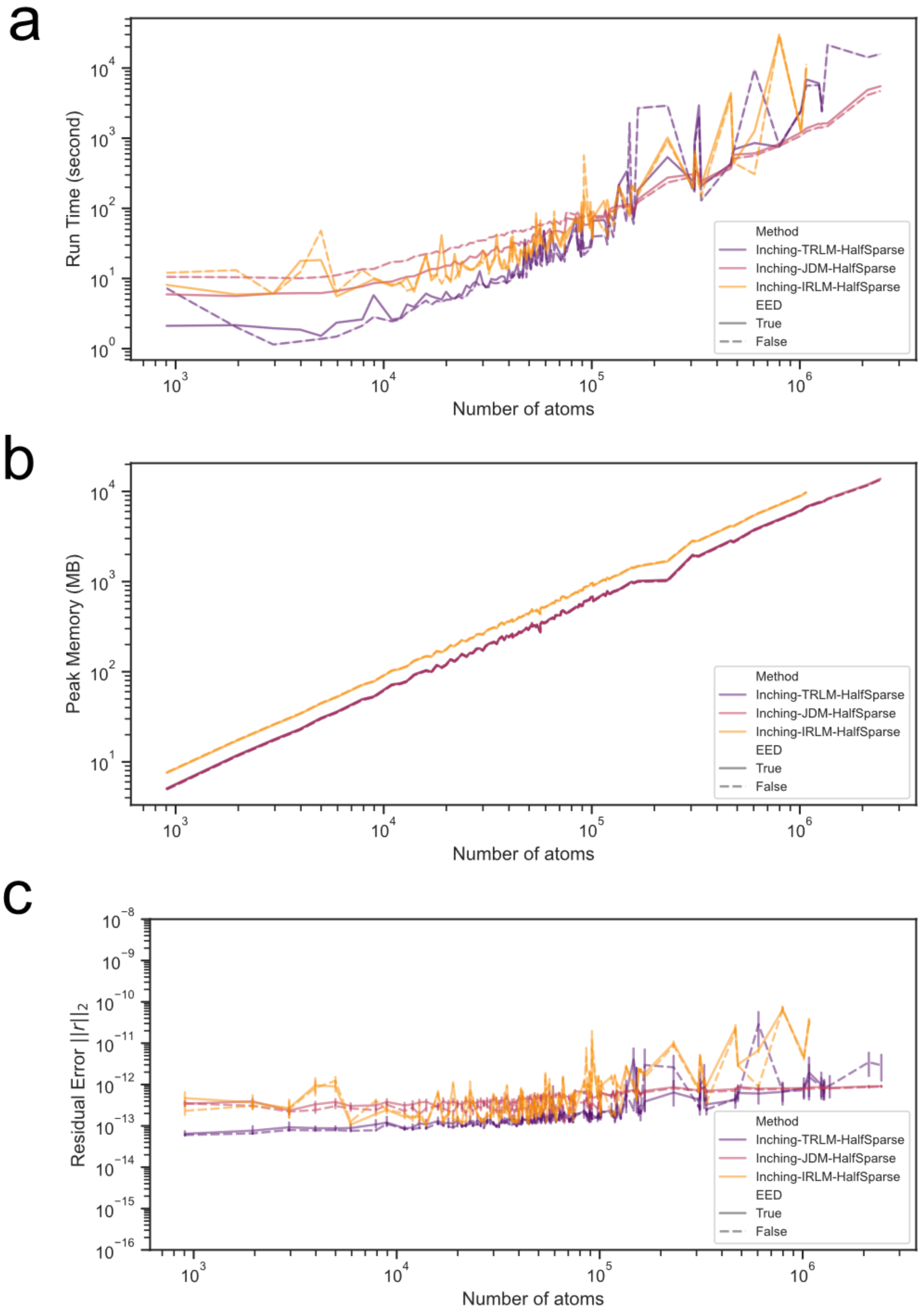

a
Run Time (second)
Number of atoms
Method
Inching-TRLM-HalfSparse
Inching-JDM-HalfSparse
Inching-IRLM-HalfSparse
EED
True
False
b
Peak Memory (MB)
Number of atoms
Method
Inching-TRLM-HalfSparse
Inching-JDM-HalfSparse
Inching-IRLM-HalfSparse
EED
True
False
c
Residual Error $||r||_2$
Number of atoms
Method
Inching-TRLM-HalfSparse
Inching-JDM-HalfSparse
Inching-IRLM-HalfSparse
EED
True
False

Supplementary Figure 6. Comparison of the effect of explicit external deflation (EED) on throughput, memory consumption and correctness in the benchmark dataset. For all methods, the first 64 eigenpairs were calculated with radius R_C = 8 Å. Methods tested includes 3 INCHING methods (i.e. INCHING-TRLM-HalfSparse, INCHING-JDM-HalfSparse, INCHING-IRLM-HalfSparse without EED in dash line and with EED in solid line. The methods in dash line (i.e. INCHING-TRLM-HalfSparse, INCHING-JDM-HalfSparse, INCHING-IRLM-HalfSparse without EED were also displayed in Figure 2 of main text. In all methods, "Half" means only the lower triangle of the Hessian matrix is accessed and stored and the storage format of the Hessian matrix is always "Sparse" meaning a Compressed Sparse Row (CSR) format is stored in double precision. INCHING-TRLM-HalfSparse, INCHING-JDM-HalfSparse, INCHING-IRLM-HalfSparse are our implementation of the Thick Restart Lanczos Method (TRLM), Jacobi-Davidson Method (JDM) and Implicitly Restarted Lanczos Method (IRLM). (a) Benchmark on overall run time including Hessian realization and subsequent diagonalization. The run time is wall-clock time to complete all the calculation. (b) Benchmark on peak memory consumption. Also note that INCHING-TRLM-HalfSparse and INCHING-JDM-HalfSparse share very similar peak memory requirement. (c) Benchmark on residual error. The error bar presented is the 95% confidence interval (n=64) calculated from the residual error of all the eigenvalues of a macromolecular structure in the benchmark dataset. All programs were stopped if run time exceeds 48 hours. All INCHING programs were stopped if number of restarts exceeded 15000 rounds.

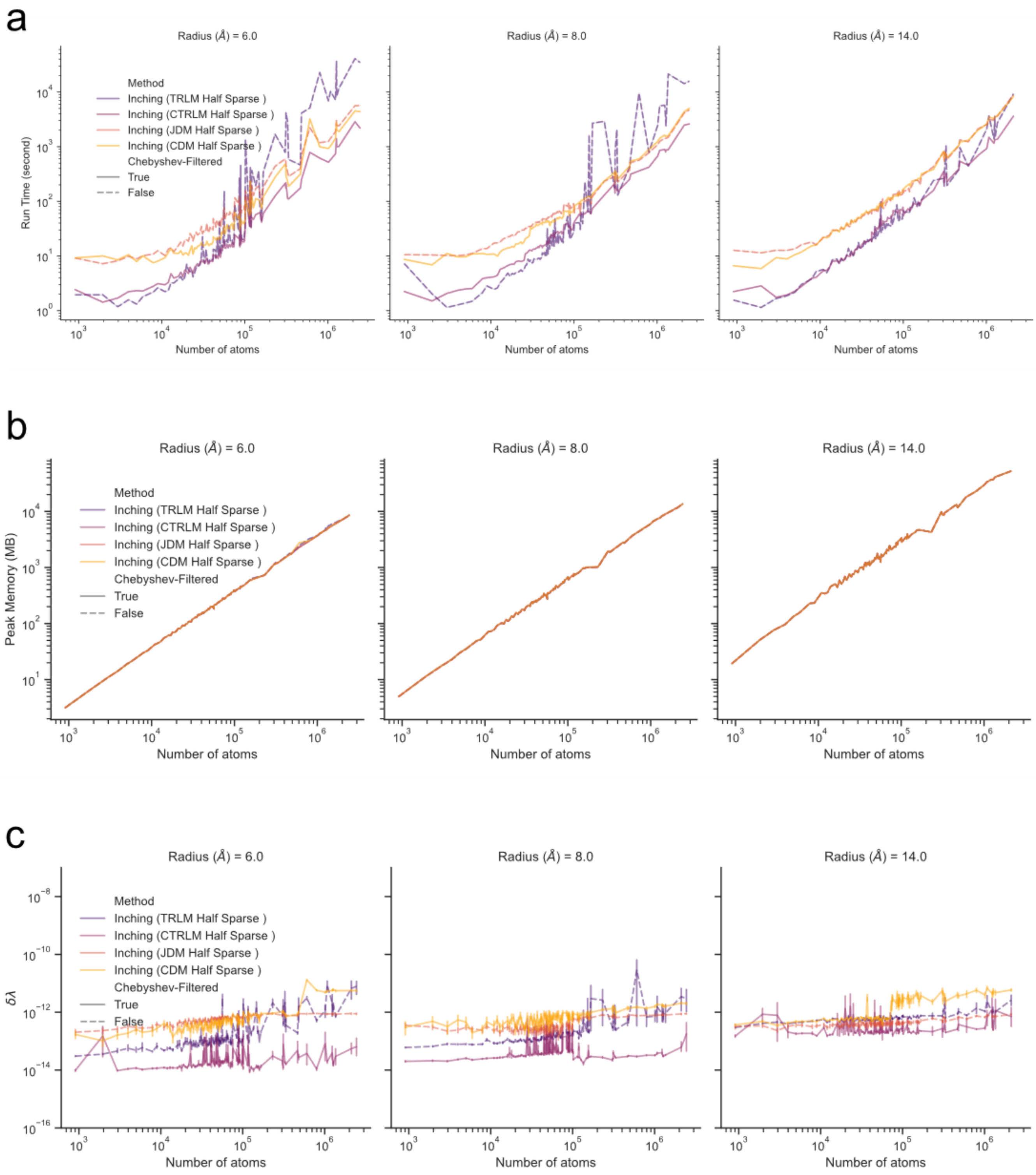

Supplementary Figure 7. Scaling in cutoff radii and effect of Chebyshev filtering on throughput, memory consumption and correctness in the benchmark dataset. For all methods, the first 64

eigenpairs were calculated. Methods tested includes 4 INCHING methods (i.e. INCHING-TRLM-HalfSparse, INCHING-JDM-HalfSparse, INCHING-CTRLM-HalfSparse and INCHING-CDM-HalfSparse. Methods with Chebyshev low-pass filtering in solid line, otherwise dashline. INCHING-TRLM-HalfSparse, INCHING-JDM-HalfSparse and INCHING-CTRLM-HalfSparse were also displayed in Figure 2 of main text. In all methods, "Half" means only the lower triangle of the Hessian matrix is accessed and stored and the storage format of the Hessian matrix is always "Sparse" meaning a Compressed Sparse Row (CSR) format is stored in double precision. INCHING-TRLM-HalfSparse, INCHING-JDM-HalfSparse, INCHING-CTRLM-HalfSparse and INCHING-CDM-HalfSparse are our implementation of the Thick Restart Lanczos Method (TRLM), Jacobi-Davidson Method (JDM), Chebshev-filtered Thick Restart Lanczos Method (CTRLM) and Chebyshev-Davidson Method (CDM). Three cutoff radii were considered including R_C = 6 Å,8 Å and 14 Å. (a) Benchmark on overall run time including Hessian realization and subsequent diagonalization. The run time is wall-clock time to complete all the calculation. (b) Benchmark on peak memory consumption. (c) Benchmark on residual error. The error bar presented is the 95% confidence interval (n=64) calculated from the residual error of all the eigenvalues of a macromolecular structure in the benchmark dataset.

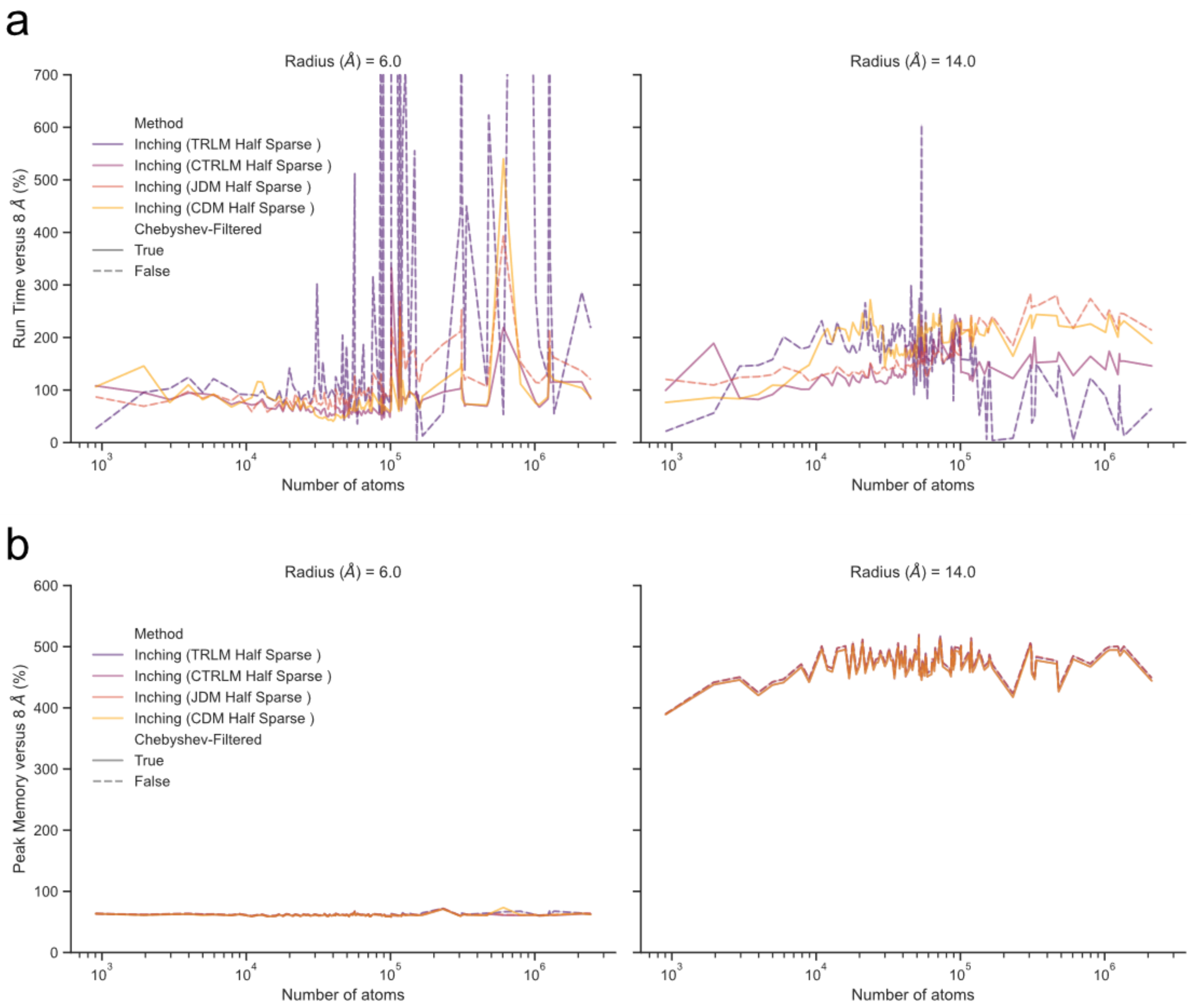

Supplementary Figure 8. Comparison of 6 Å and 14 Å calculations. Comparison in the percentage of run time (a) and peak memory (b) versus standard 8 Å calculations. Note that comparison of scaling in radii in memory is dependent on the number and indexing of non-zero entries in Hessian, where for all 14 Å calculations, 64-bit indexing were used instead of 32-bit indexing to accommodate the growth in non-zero entries in Hessian beyond capability of 32-bit indexing. For all methods, the first 64 eigenpairs were calculated. Methods tested includes 4 INCHING methods (i.e. INCHING-TRLM-HalfSparse, INCHING-

JDM-HalfSparse, INCHING-CTRLM-HalfSparse and INCHING-CDM-HalfSparse. Methods with Chebyshev low-pass filtering in solid line, otherwise dashline. INCHING-TRLM-HalfSparse, INCHING-JDM-HalfSparse and INCHING-CTRLM-HalfSparse were also displayed in Figure 2 of main text. In all methods, “Half” means only the lower triangle of the Hessian matrix is accessed and stored and the storage format of the Hessian matrix is always “Sparse” meaning a Compressed Sparse Row (CSR) format is stored in double precision. INCHING-TRLM-HalfSparse, INCHING-JDM-HalfSparse, INCHING-CTRLM-HalfSparse and INCHING-CDM-HalfSparse are our implementation of the Thick Restart Lanczos Method (TRLM), Jacobi-Davidson Method (JDM), Chebshev-filtered Thick Restart Lanczos Method (CTRLM) and Chebyshev-Davidson Method (CDM). Three cutoff radii were considered including R_C = 6 Å,8 Å and 14 Å.

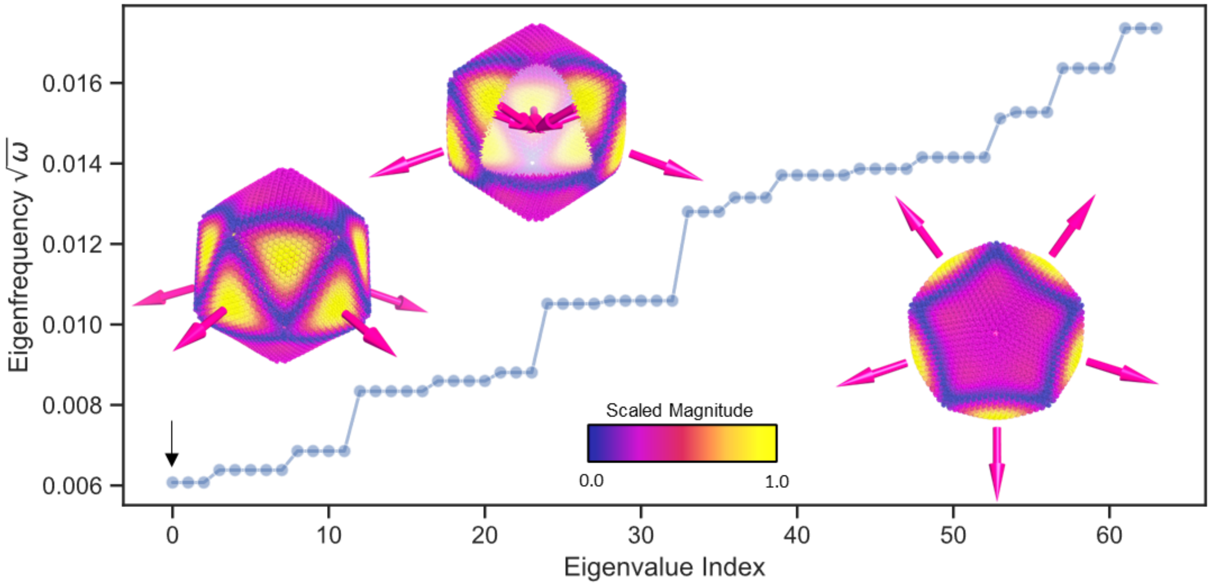

Supplementary Figure 9. The first 64 eigenfrequency of the Faustovirus capsid (PDBID: 5J7V) coarse-grained as 5 million pseudoatoms. Inset shows the mode shapes of the first non-rigid mode. The left- and right-most insets are sideview and bird-eye view respectively; the middle inset is the sideview sectioned to show pattern inside. The arrow in magenta indicates an average direction for local clusters of the displacement field.

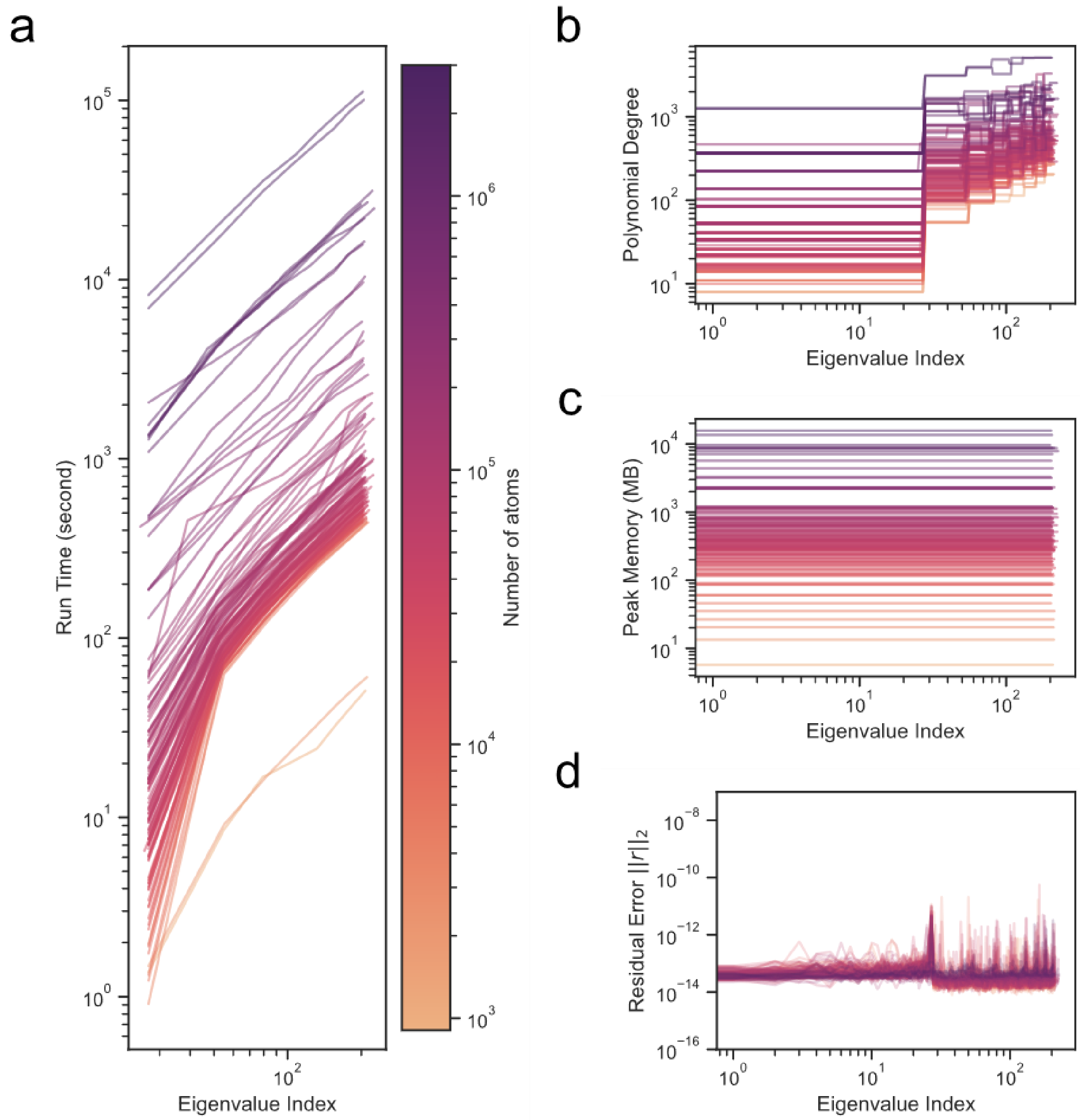

Supplementary Figure 10. Further Results in applying Chebyshev-filtered Thick Restart Lanczos Method. Scaling in number of eigenmodes on GPU RTX4090 in batches of 28 eigenvectors. Up to 200 or slightly more eigenpairs were calculated using INCHING-CTRLM-HalfSparse with cutoff radius R_C = 8Å. The coloring of the lines, as shown in the color bar, refers to the number of atoms in the system. (a)

Run time to complete until the indexed eigenvalue, (b) optimized polynomial degrees involved in calculating the indexed eigenvalue, (c) peak memory consumed when calculating the spectrum slice containing the indexed eigenvalue and (d) residual error in each of the eigenvalues were also shown.

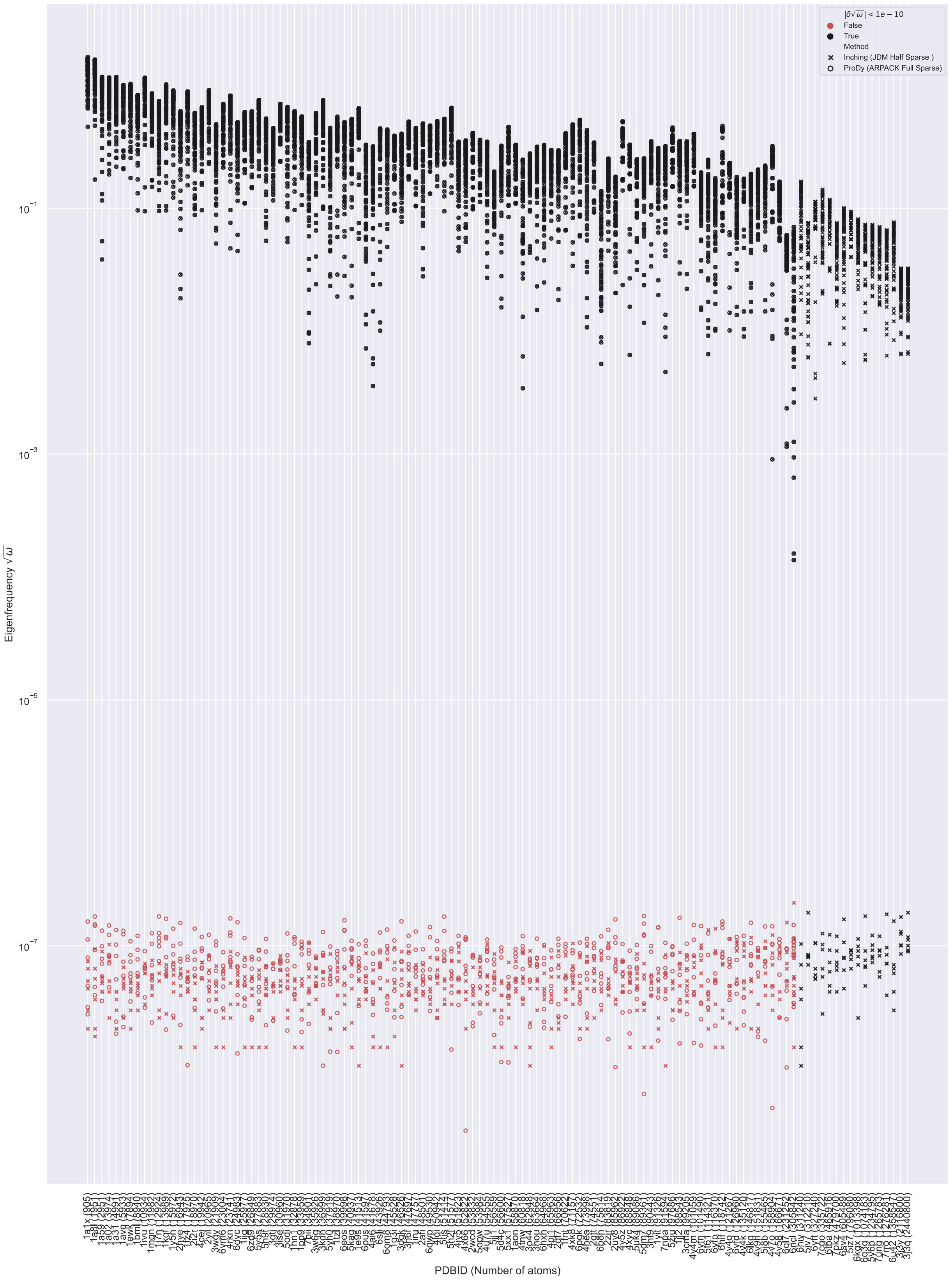
$|\delta\sqrt{\omega}| < 1e-10$
False
True
Method
Inching (JDM Half Sparse)
ProDy (ARPACK Full Sparse)
10^{-1}
10^{-3}
10^{-5}
10^{-7}
Eigenfrequency $\sqrt{\omega}$
PDBID (Number of atoms)

Supplementary Figure 11. Comparing Eigenfrequencies calculated. Eigenfrequencies computed with INCHING-JDM-HalfSparse in cross marker, compared to those from ProDy with ARPACK backend, i.e. ProDy-ARPACK-FullSparse in circle marker. The ARPACK package is a golden standard implementation of the Implicitly Restarted Lanczos Method (IRLM). Whenever the eigenvalue of the same index calculated by INCHING-JDM-HalfSparse differs from ProDy-ARPACK-FullSparse with absolute value larger than 10^{-10}, the difference is highlighted in red. For cases where ProDy-ARPACK-FullSparse did not converge within 48 hours, there is no datapoint. In general, eigenfrequencies less than 10^{-6} are considered rigid modes with eigenvalue zero. In all cases considered, there are only 6 rigid modes. For calculation of eigenfrequencies, absolute value was applied to all eigenvalues to avoid spurious imaginary eigenfrequencies for rigid modes with small negative numerical eigenvalue, e.g., -10^{-14}. For all methods, the first 64 eigenpairs were calculated with radius R_C = 8 Å.
