# Supplementary material for: Scalable computation of anisotropic vibrations for large macromolecular assemblies
Source: Nat Commun. 2024 Apr 24;15:3479. doi: 10.1038/s41467-024-47685-8 (PMC11043083; doi:10.1038/s41467-024-47685-8)
Supplement: Supplementary file 3 — Description of Additional Supplementary Files [file 41467_2024_47685_MOESM3_ESM.docx]

**Description of Additional Supplementary Files**

**Supplementary Movie 1:** The first non-rigid vibrational mode of a mature HIV-1 capsid structure (PDBID:3J3Q)

**Supplementary Movie 2:** The first non-rigid vibrational mode of a ribosome bound to elongation factor G (PDBID:4V9H)
