# Supplementary figures and images for: Scalable computation of anisotropic vibrations for large macromolecular assemblies
Source: Nat Commun. 2024 Apr 24;15:3479. doi: 10.1038/s41467-024-47685-8 (PMC11043083; doi:10.1038/s41467-024-47685-8)

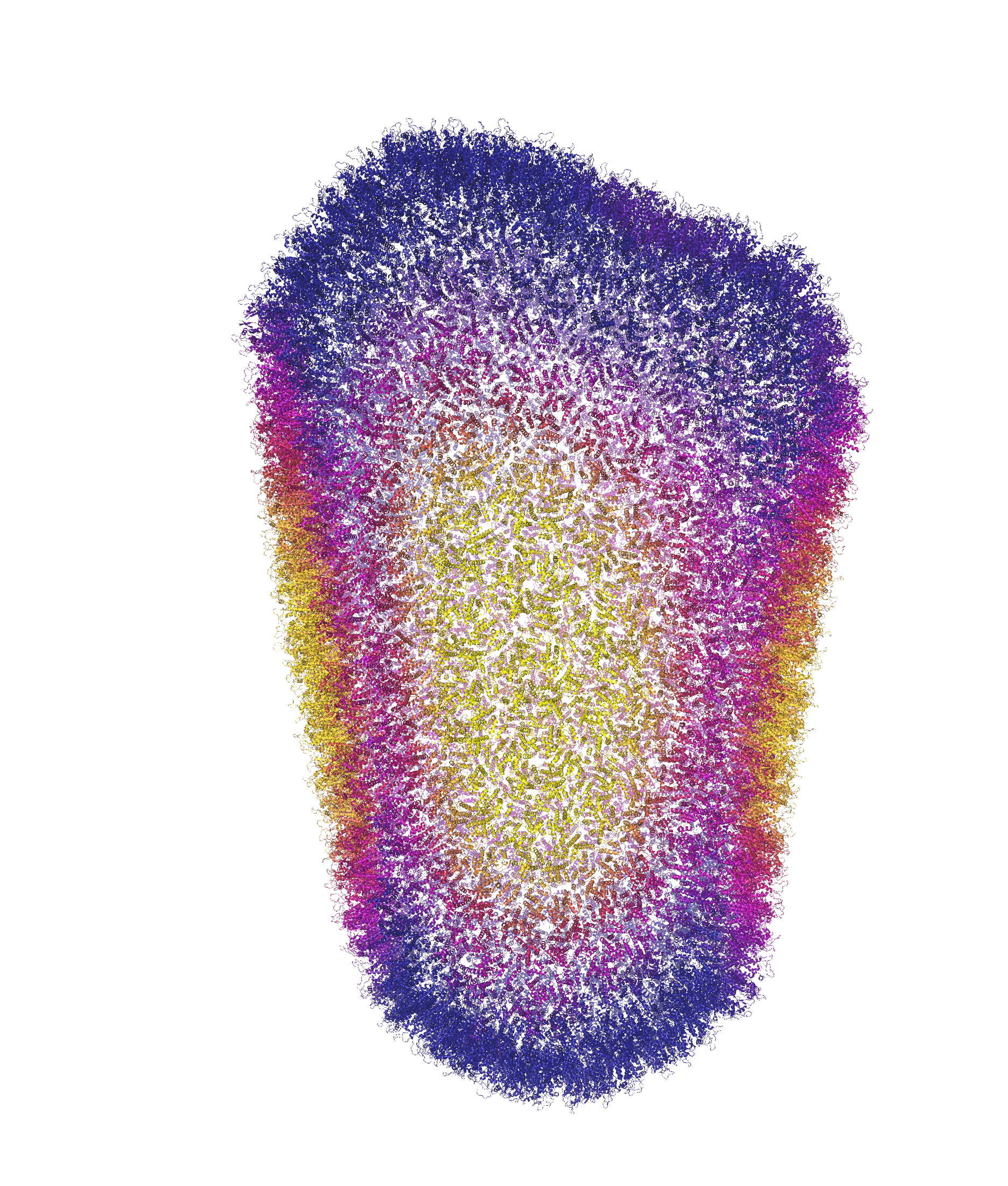

Supplement: Supplementary file 4 — Supplementary Movie 1 [file 41467_2024_47685_MOESM4_ESM.gif]

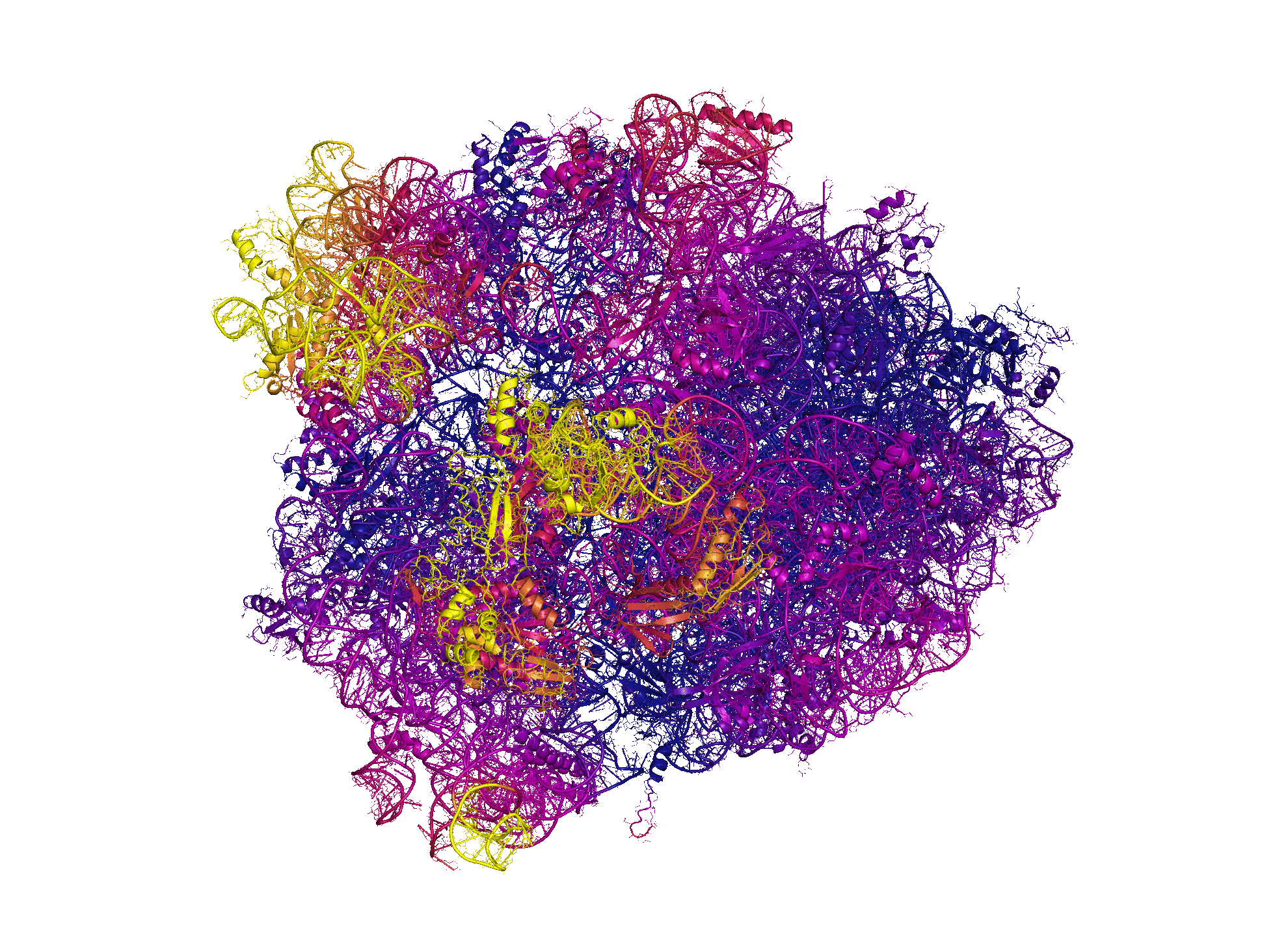

Supplement: Supplementary file 5 — Supplementary Movie 2 [file 41467_2024_47685_MOESM5_ESM.gif]
